# The Viscoelastic Properties of Passive Eye Muscle in Primates. II: Testing the Quasi-Linear Theory

**DOI:** 10.1371/journal.pone.0006480

**Published:** 2009-08-03

**Authors:** Christian Quaia, Howard S. Ying, Lance M. Optican

**Affiliations:** 1 Laboratory of Sensorimotor Research, National Eye Institute, Bethesda, Maryland, United States of America; 2 Wilmer Eye Institute, Department of Ophthalmology, The Johns Hopkins Hospital, Baltimore, Maryland, United States of America; University of Arizona, United States of America

## Abstract

We have extensively investigated the mechanical properties of passive eye muscles, *in vivo*, in anesthetized and paralyzed monkeys. The complexity inherent in rheological measurements makes it desirable to present the results in terms of a mathematical model. Because Fung's quasi-linear viscoelastic (QLV) model has been particularly successful in capturing the viscoelastic properties of passive biological tissues, here we analyze this dataset within the framework of Fung's theory.

We found that the basic properties assumed under the QLV theory (separability and superposition) are not typical of passive eye muscles. We show that some recent extensions of Fung's model can deal successfully with the lack of separability, but fail to reproduce the deviation from superposition.

While appealing for their elegance, the QLV model and its descendants are not able to capture the complex mechanical properties of passive eye muscles. In particular, our measurements suggest that in a passive extraocular muscle the force does not depend on the entire length history, but to a great extent is only a function of the last elongation to which it has been subjected. It is currently unknown whether other passive biological tissues behave similarly.

## Introduction

The first extensive study of muscle as a viscoelastic material (i.e., using the analytic methods of rheology) was carried out on single fibers and small bundles of fibers from frog skeletal striated muscles [1]. As a way of synthetically summarizing their results, Buchthal and Kaiser fit a separate linear model to each force transient induced by a small stepwise change in muscle length. The parameters of the model varied (nonlinearly) as a function of initial muscle length, but were kept constant during any one simulation. No single nonlinear model able to reproduce all the data with one set of parameters was presented. More recently, several extremely accurate studies have been conducted to investigate the properties of individual skeletal muscles fibers, mainly in frogs (e.g., [2]–[4]) and in rats (e.g., [5]–[9]). However, the modeling was conducted along the lines of Buchthal and Kaiser, i.e., using a set of locally linear models. Models like these are certainly valuable, as they summarize the data and enable comparisons across different datasets. However, they have no predictive power, because they cannot be used to simulate the force induced by a generic elongation. This is obviously an important limitation.

**Figure 1:**
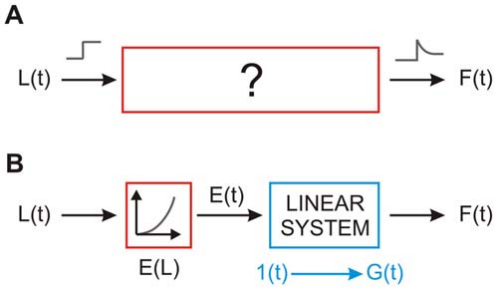
Schematic view of Fung's theory. A: In most biological passive materials, a stepwise change in elongation (gray trace on the left) causes the force to suddenly increase and then decay over time (gray trace on the right). B: Fung proposed that this response scales with the size of the step, and that there is a nonlinear relationship E(L) between the peak force and the step size (which he called elastic response). He also posited that the decaying response is generated by a linear system, which can be described in terms of its step response. G(t) is then the response of the linear subsystem to a unitary step, 1(t), of the elastic response. This is a cascade of a static nonlinearity and a linear system, often referred to as a Hammerstein system. The model is not limited to reproducing the response to a strain step, but can be used to predict the response to an arbitrary strain history. Blue blocks indicate linear processes, whereas red blocks indicate the presence of a nonlinearity.

To find more comprehensive models, one needs to turn to studies of biological tissues composed mostly of collagen. A particularly successful attempt to model the nonlinear viscoelastic properties of passive tissues was carried out by Fung [10]. Fung measured the force *F* following a stepwise change in the length *L* of a section of rabbit mesentery membrane, starting from a resting condition. He concluded that the time course of this response scales nonlinearly with the magnitude of the step, and it can thus be modeled as
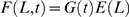
(1)where *F(L, t)* is the relaxation response, *G(t)* is what he called the *reduced relaxation function* (normalized so that *G(0)* = 1), and *E(L)* is the *elastic response*, i.e., the force instantaneously generated in the tissue when the length is changed in a stepwise manner from the resting length to L. That is, he posited that the relaxation response is *separable*. Next, Fung assumed that the *superposition principle* holds, so that the response to a generic elongation history can be interpreted as the infinite sum of relaxation responses to infinitesimally small step-like changes in the elastic response. By doing so he essentially assumed that the elastic response is responsible for the nonlinear behavior, whereas the reduced relaxation function is generated by a linear viscoelastic process that acts on that elastic response. Accordingly, the nonlinear process that converts the elongation into the force (Fig. 1A) is interpreted by Fung (Fig. 1B) as the cascade of a static nonlinearity (the elastic response, red box) followed by a linear process (with step response *G(t)*, blue box). This model was dubbed *quasi-linear viscoelastic* (QLV), and it has since been applied to describe the viscoelastic behavior of a wide range of biological materials, such as tendons, ligaments, veins, arteries, cartilage, cardiac and skeletal muscle (e.g., [11]–[31]). Note that this model is fit to the data using the response to step-wise elongations but, unlike those mentioned in the previous paragraph, it can then in principle be used to predict the response to a generic elongation.

The elegance and wide success of the quasi-linear framework make it an ideal candidate for our object of inquiry, passive extraocular muscles (EOMs). The purpose of this study is thus to apply this theory to the length-tension data we have collected in passive EOMs. However, the underlying assumptions of the QLV model have rarely been systematically tested, and recent reports indicate that, at least in ligaments [25], [32]–[34] and in reconstituted collagen [35], [36], the separability hypothesis embedded in the quasi-linear model does not hold. We designed our experiments accordingly, carrying out a comprehensive test of the quasi-linear theory (within the limits imposed by our experimental preparation). We found that the original QLV model is unable to reproduce the forces generated by passive eye muscles in response to stepwise changes in length (within the physiological range). Extensions of this model, notably the “adaptive quasi-linear model” [36], are able to overcome some of the problems, but not others. This raises the possibility that the QLV model and its descendants might be less than ideal to approximate the behavior of other passive biological materials as well.

## Methods

The methods used to collect the data presented in this paper have been described in great detail in the previous paper in this series [37]. Here, only a brief summary is provided.

### Ethics Statement

All procedures were in agreement with the Public Health Service policy on the humane care and use of laboratory animals and all protocols were approved by the Animal Care and Use Committee of the National Eye Institute.

### Animals

Eye muscle forces were measured in three adult rhesus monkeys (*Macaca mulatta*), ranging in weight from 8 to 14 Kg (identified as m2, m3, and m4). None of the animals had been previously used in any experiment, and their eyes and orbits were thus pristine.

### Surgical procedure

The animal was placed supine on the surgical table, intubated, anesthetized with isoflurane (2–4%) in oxygen, and mechanically ventilated. Heart rate, indirect mean arterial blood pressure, mucus membrane color, peripheral oxygenation/SpO_2_, end-expiratory CO_2_ partial pressure, and EKG were monitored and maintained within normal physiological ranges. Body temperature was monitored and maintained at 37°C with a heating pad. Paralysis was induced with pancuronium bromide (0.05–0.10 mg/Kg IV), and was maintained by administering a reduced dose (0.025–0.050 mg/Kg IV) every 45 minutes until the end of the procedure. The paralytic agent was used to ensure that the muscles were completely passive. At the end of the procedure, and while still deeply anesthetized, the animal was euthanized with an overdose of sodium pentobarbital (150–250 mg/kg).

### Experimental procedure

After the animal had been anesthetized, its head was stabilized with a stereotaxic device's ear bars (to reduce the head's degrees of freedom from six to one). A mouth bar added to the stereotaxic device was attached to the front teeth with dental cement to fix the head so that Reid's baseline was perpendicular to the table. Both eyes were prepped and draped in the usual sterile ophthalmic manner. The conjunctiva was then incised in correspondence with an eye muscle insertion on the globe, and a muscle hook was placed under the insertion. From here we adopted two different techniques.

In four muscles (identified as m2SR, m3LR, m3SR, and m4MR), the muscle was connected to the measuring device directly by a Kevlar™ thread (between 50 and 75 mm long). The connection was achieved by sandwiching the wire, together with the tendon, between two tiny titanium plates (6 mm by 2 mm by 1 mm) kept together by two microscrews (total weight 0.05 g). The stiffness of the connection was between 5400 and 8100 gf/mm. On the last muscle tested (identified as m4LR), we did not use the above described clamping technique, but instead tied a Surgidac™ (US Surgical) 5-0 surgical suture to the tendon and then knotted its other end to the distal end of the Kevlar wire (the knot was then secured with a very small metallic crimp, weight 0.02 g). The overall stiffness of the Surgidac-Kevlar connection was 2450 gf/mm.

Muscle force was measured using an Aurora Scientific (Aurora, ON, Canada) 305C Dual-Mode Muscle Lever System. In the experiments described here we imposed the muscle length, and measured the corresponding change in force (NB: the SI standard unit of force is the Newton (N), but muscle force is traditionally measured in units of *gram force* (1 gf≈0.0098 N); e.g., a mass of 102 g exerts a force of 102 gf, or 1 N, on earth). The specifications for the system used are as follows:

- Length Signal Resolution: 1 micron
- Length Signal Linearity: 0.1% over the center 4 millimeters, 0.5% over the entire 20 mm range
- Length Step Response Time (1% to 99% critically damped): 2.0 msec
- Sinusoidal Frequency Response (-3dB): 330 Hz
- Force Signal Resolution: 1.0 mN (∼0.1 gf)
- Force Signal Linearity: 0.2% of force change

Both the length and the force signals are low-pass filtered with a 4^th^ order Butterworth filter with a cut off frequency of 5 kHz. The bandwidth of the system is limited by the motion bandwidth, not by the sensor bandwidth. In all our experiments we stayed well within the bandwidth of the equipment. In doing so we guaranteed that the measurement device was not a limiting factor, and that both the length and force sensor outputs can be treated as veridical. The input/output analog signals for/from this device were generated and acquired through an A/D-D/A interface board (National Instruments, NI USB-6211) connected to a laptop PC (IBM, Amonk, NY) and controlled by LabView (National Instruments, Austin, TX). The experiment was controlled by a custom Java program that communicated with LabView, displayed the data in real-time, and stored it for later analysis.

Based on the results and modeling studies from other passive biological tissues we concluded that, to conduct a thorough test of the quasi-linear model, the minimum set of experimental paradigms to be applied was the following:

- Small elongation steps, executed within a few milliseconds, from initial lengths spanning the entire elongation range tested.
- Sequences of double steps, separated by variable time intervals (0.01, 0.1, 1, and 45 s), from initial lengths spanning the entire elongation range.

All the steps we imposed had an amplitude of 0.5 mm. In all muscles we used steps that had a peak speed of 160 mm/s, a peak acceleration/deceleration of 144 mm/s^2^, and a duration of 4.5 ms (bandwidth 130 Hz, Welch's method). In some muscles we also induced some slower steps, with a peak speed of 80 mm/s, a peak acceleration/deceleration of 74 mm/s^2^, and a duration of 8 ms (bandwidth 50 Hz). Long waiting periods were imposed before and after each length change. In addition, we performed constant-speed ramps spanning the entire elongation range, at various speeds (1, 10, 80, and 160 mm/s). Other paradigms were also used in the experiments, but they will be described and analyzed in a subsequent paper.

Only lengthening was tested, because it was technically impossible for us to measure the forces during shortening (they become negative for even relatively low shortening speeds). Another limitation of our study is that we never exceeded a one-hour testing period per muscle, as we wanted to avoid any tissue deterioration. Hence, because after each muscle elongation we waited for a long time (30 seconds in the first two monkeys, 45 s in the third) for the force to settle, we could not perform all experiments in all muscles.

The elongation range was determined separately for each muscle. The range tested always covered the entire oculomotor range (i.e., the set of lengths that are achieved in physiologic conditions, which in monkeys correspond to approximately ±45° of rotation), but never exceeded it by more than one mm. Accordingly, the elongation range tested was always about eight mm. Before recording we preconditioned the muscles by repeatedly (5–10 times) stretching and releasing them sinusoidally over their entire range (which is standard procedure in tissue rheology to guarantee repeatable results; the relatively low number of cycles used here is justified by the *in vivo* condition we used). For all muscles tested, we ran a block of three-four ramps at the beginning and at the end of the experiment to test for any possible deterioration of the muscle. We never observed any significant change in these test trials.

In our experimental preparation, the raw force measures are affected by a significant heartbeat and respiration-related noise. As explained at length in the first paper in this series [37], we devised a method to very effectively remove, *post hoc*, both of these noise components. The residual measurement noise was extremely small, at or below the level of our instrumentation accuracy.

### Simulations

The models presented in this article (described by Eqs. 13 and 20) were simulated numerically in Matlab™ (The Mathworks, Natick, MA). The scripts are available upon request. Parameter optimization for the QLV model was carried out using a commercial optimization package (modeFRONTIER™, Esteco s.r.l., Trieste, Italy).

## Results

In the previous paper in this series [37] we described measurements of the mechanical properties of passive extraocular muscles in monkeys. More precisely, we reported analytic fits for the static length-tension curve (i.e., the curve that describes the relationship between muscle length and the steady-state force exerted by the passive muscle at that length) and for the relaxation response (i.e., the force measured following a small step-wise change in elongation). These were only descriptive fits, and no attempt at modeling the data was made.

Here we 1) fit two models (developed by others) to the data set reported in our previous paper, and 2) test, using additional experimental data, the underlying assumptions of those models.

### Applying the QLV model to passive eye muscles

As noted in the Introduction, the QLV model has two major components, which can be fit separately to the data. In principle, the *elastic response* (*E(L)* in Eq. 1) should be determined by measuring the force exerted right after an instantaneous step. Because it is not possible to execute an instantaneous step, we clearly cannot follow this procedure. However, under the QLV theory the reduced relaxation function *G(t)* is assumed to be independent of length, implying that long after a step (i.e., at equilibrium) the following equation will always hold:
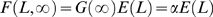
(2)where *G(∞)* = α is the ratio between the asymptotic force after a step and the force immediately after an (instantaneous) step (remember that *G(0)* = 1). By definition *F(L,∞)* is the static length-tension relationship, which we have described and quantified in the previous paper in this series [37], identifying it as *T(L)*. It thus follows that
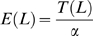
(3)


Because α cannot be measured, it must be estimated. However, Eq. 3 tells us that the dependency of the elastic response on length is already embedded in the length-tension curve. This is a special case of the more general observation that in the QLV model any isochronal stress-strain relationship is proportional to the elastic response [23], [34].

We found that in passive eye muscles the length-tension relationship is well fit by the following expression:
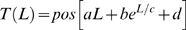
(4)where *pos[ ]* indicates that negative values are truncated to zero. *E(L)* will thus have the same form, which is somewhat different than those commonly encountered in the QLV literature [30].

The second crucial component in Fung's QLV theory is the *reduced relaxation* function *G(t)*, which describes the time course of the force induced by a unitary step in the elastic response. The exact form of *G(t)* is not prescribed by the QLV theory, and several expressions, such as those based on a continuous exponential spectrum [38], [39], on fractional derivatives [40], or on variable-order differential operators [41], have been used over the years. In our context the form based on a discrete exponential spectrum is the natural choice (it will become clear why shortly).

Accordingly, we define *G(t)* as
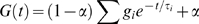
(5)with
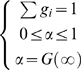



Once a form for *G(t)* has been chosen, the value of each parameter must be inferred from the data (i.e, the model has to be fit to the data). Here we can take advantage of the fact that in our previous paper [37] we already provided a fit for the relaxation response measured following a small incremental step in elongation. For a step in which the muscle length is varied from L_0_ at t = 0 to L_0_+ΔL at t = t_0_, and the final length is maintained afterwards, we defined the following fit
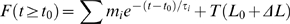
(6)where again *T(L)* is the static (i.e., steady-state) force-length relationship.

Using a procedure that we developed [37], we found a set of seven time constants *τ_i_* that was compatible with the signal to noise ratio in our dataset. We then applied, independently for each step, the Emri-Tschoegl algorithm [42]–[45] to find the moduli *m_i_* that yielded the best fit to the data. Eq. 6 allowed us to fit the relaxation response extremely well. It should now appear clear why we chose Eq. 5 for *G(t)*: since they are both sums of exponentials, it should be fairly easy to find the parameters for Eq. 5Eq. 5 from our previous fits.

There is however one major caveat: in the QLV model there is a single reduced relaxation function, whereas we have independent fits at each elongation. Since there is no rational way of selecting one fit over another, we will instead determine the parameters for what has been called the *generalized QLV model* [35], [36]. In this model the reduced relaxation function can vary as a function of elongation (i.e., separability does not hold), but it is considered to be fixed over small stretches. Essentially, instead of fitting one single QLV model to the entire data set, we will start by fitting a separate QLV model to each elongation step.

We mentioned in the Introduction how Fung's theory models the force induced by an arbitrary elongation in terms of a Boltzmann integral. More precisely, assuming that the muscle has settled and a length change is applied starting at time 0, the force *F* is expressed as
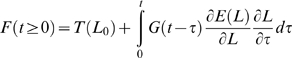
(7)where L_0_ is the initial (not necessarily resting) length.

If a quick (but not ideal) elongation step is applied, so that the length is varied from L_0_ at t = 0 to L_0_+ΔL at t = t_0_, and the final length is maintained afterwards (i.e., *dL/dt = 0, t>t_0_*), we have that 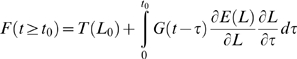
(8)


Note that Eq. 8 and Eq. 6 describe the same force, and thus must be equal if we want to fit the model to the data. Because the steps we used are relatively small and quick, we can approximate this equation by positing that 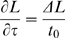
for 0≤t≤t_0_, and is 0 for all other times. Similarly, if the steps are small enough we can locally linearize the elastic response, and assume that 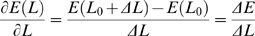
for 0≤t≤t_0_, and is 0 for all other times. Because 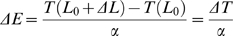



Eq. 8 yields
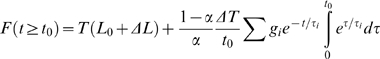
(9)


If we now define
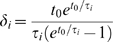
(10)it is easy to show that Eqs. 6 and 9 are equal at all times if and only if:
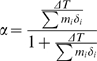
(11)and
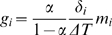
(12)


Note that δ_i_ is equal to 1 when τ_i_ is large relative to t_0_, and so for large time constants the *g_i_* values are simply a scaled version of the corresponding *m_i_* values. With an ideal (i.e., instantaneous) step this would be true for all time constants. Similar procedures have been developed to deal with the more commonly used continuous spectrum form of *G(t)* [18], [46].

**Figure 2:**
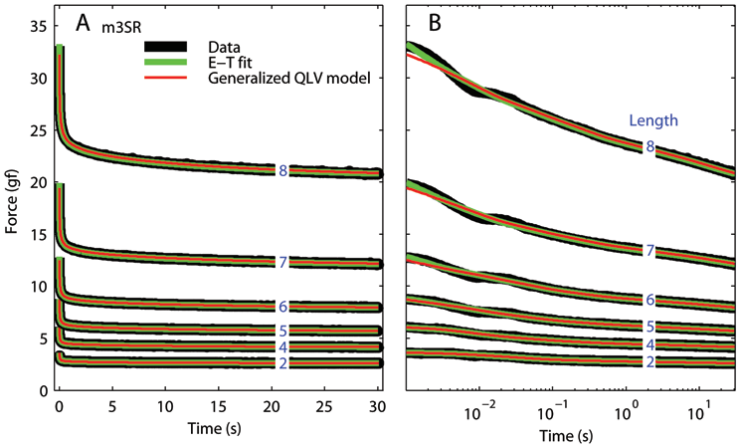
Relaxation responses and generalized QLV model fits. A: Data (black), Emri-Tschoegl fit to the relaxation response (green), and force predicted by the generalized QLV model using the parameters derived analytically from the E-T fit (red). Six different steps are shown (blue numbers are the final lengths in mm), from the superior rectus in m3. B: Same as A, but using a logarithmically spaced abscissa to improve visualization of the force at short times.

Our approximation yields estimates of *E(L)* and *G(t)* that, when plugged into Eq. 8, produce a very good fit to the data *after* the end of each step elongation (Fig. 2). However, because of the approximations and assumptions introduced above, and because of the finite duration of the step, the resulting model cannot be expected to perfectly reproduce the data *during* the elongation phase. Furthermore, there is evidence in our data for a purely viscous component [37], which obviously cannot be reproduced by the QLV model. Accordingly, we extended the (generalized) QLV model by adding a purely viscous term:
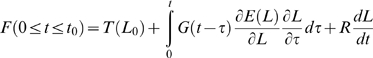
(13)


We then directly fitted Eq. 13 to the force during the elongation. We reasoned that only the moduli for the two shortest time constants could have been significantly affected by the above described approximations. We thus considered R and the first two moduli *m_1_* and *m_2_* as parameters (with the values produced by the Emri-Tschoegl algorithm as initial guesses for the *m_i_*), and conducted an optimization search to find the values that would yield (through Eqs. 11, 12, 5, and 13) the best possible fit to the peri-elongation data. This general approach, based on fitting the Boltzmann integral directly to the data, has been pioneered by others [31], [47], but instead of fitting all the model parameters to the data we only used it to refine a very good initial estimate. We think that this limited use of the optimization, similarly employed by others [23], [36], has a clear advantage: the initial fit to the relaxation response reduces dramatically the dimensionality of the design space, and provides good initial guesses for the parameters, reducing the computation time and making the optimization more likely to identify the best set of parameters.

We noted that, in all muscles, the viscous factor R was a function of length: it had a constant value at short elongations, and it decreased all the way to zero at large elongations. We found that this behavior could be captured very well by the following sigmoidal relationship:
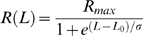
(14)


**Figure 3:**
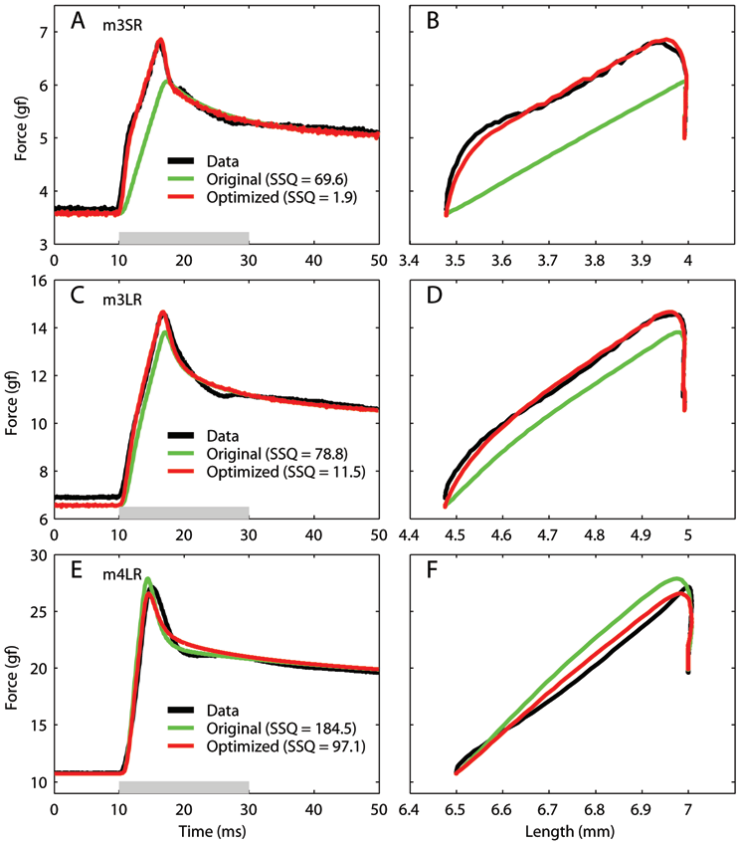
Peri-elongation forces and generalized QLV model fits. Data (black), force predicted by the generalized QLV model using the parameters derived analytically from the E-T fit (green), and force predicted by the generalized QLV model after parameter optimization and addition of a viscous term (red). A: Data for a step at short elongations from the superior rectus in m3. B: Same forces as in A, but plotted as a function of muscle elongation rather than time. The initial rapid rise in force is due to the pure viscosity, which was not part of the original model (green trace). C & D: Same as A & B, but for a step at intermediate elongations from the lateral rectus in m3. E & F: Same as A & B, but for a step at large elongations from the lateral rectus in m4. Note how in this case the fit is not as good, as the force in panel F is convex, whereas the model predictions are always concave. Force scale is different across rows. SSQ: sum of squared residuals (fit – data).

Because this parameter does not increase with length like the others, we believe that it is most likely the manifestation of an artifact, possibly friction between the muscle and surrounding tissues (which could decrease with elongation because the muscle diameter decreases as it gets stretched). In Table 1 we show, for each muscle, the values of the parameters in Eq. 14, obtained by least-squares fitting the relationship to the values of R obtained from the optimization (using as value for L the length at the end of the step). Note that the value of the viscosity is always quite small, so that the viscous force is always under 1 gf. We then ran our optimization again, this time with just *m_1_* and *m_2_* as variables, and with the estimate of the viscous force computed using Eq. 14. This algorithm worked very well on our data, leading to excellent fits to both the peri- and post-elongation epochs. In Fig. 3 we compare the forces predicted during the step (Eq. 13) using the original parameters (and no viscosity) with those obtained with the optimized parameters (and the viscous term). Note that in all cases the actual time course of the elongation *L(t)*, as reported by the position sensor, was used in the simulations (samples of the velocity profile are shown in Fig. 7 of the previous paper [37]). Three representative steps are shown, from three different muscles and at three different lengths. On the left the time-course of the force is plotted, whereas on the right the force is plotted as a function of muscle elongation. The improvement due to the optimization is obviously significant, especially at shorter muscle lengths, where the viscous force plays a prominent role. At the largest elongation (bottom row) the improvement is significant, but the optimized fit is not as good. Unfortunately this is a limit of the model, which is unable to reproduce the convexity of the length-force function (within these small elongations, the model always generates concave length-force functions).

**Figure 4:**
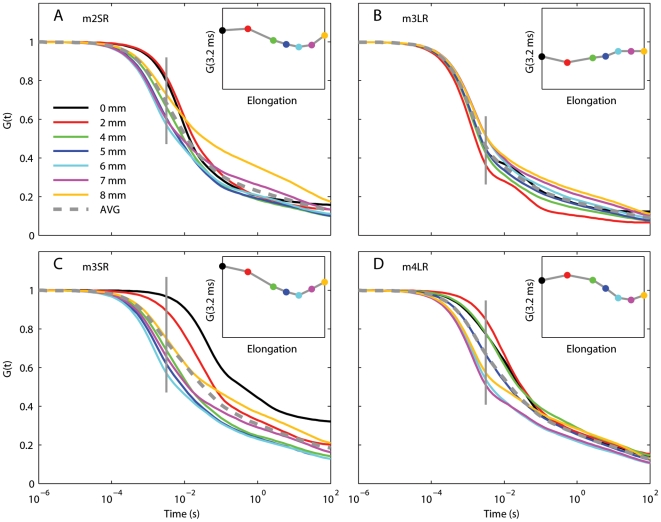
Reduced relaxation function for the generalized QLV model. In each panel we plot, as a function of time, the step response of the linear part of the QLV model (Fig. 1B). Since we used the generalized QLV model there is a curve for each muscle length. If the separability hypothesis held, these curves should all be identical. In the insets we plot the value of the reduced relaxation function at time 3.2 ms (vertical gray line in the main plots) as a function of elongation. Notice how this relationship is in all cases smooth, which would not be expected if the variations were due to random noise or fitting errors. The label in each panel indicates which monkey (m2, m3 or m4) and muscle (LR = lateral rectus, SR = superior rectus) the data are from. AVG: average reduced relaxation function (gray dashed line).

**Table 1:** Parameters for the *R(L)* function in each muscle.

	R_max_	L_0_	σ
m2SR	0.0080	6.7000	0.5018
m3LR	0.0110	7.5000	0.2224
m3SR	0.0120	7.8000	0.3070
m4LR	0.0115	5.4000	0.4874

### Testing the Separability Hypothesis

In the preceding section we have shown that the generalized QLV model is able to reproduce our step data very well. In this model a separate reduced relaxation function is fitted to the data for each step (i.e., at each muscle length tested). As we noted in the Introduction, the original QLV model rests instead on the hypothesis that the relaxation response is separable into an elastic response *E(L)* (a *nonlinear* function of muscle length) and a *length-invariant* reduced relaxation function *G(t)* (the step response of a *linear* dynamic system).

In Fig. 4 we plot, separately for each muscle, the *G(t)* functions for our generalized QLV model (one for each muscle length). Remember that these are the step responses of the linear part of the (generalized) QLV model. Obviously, if the original QLV model holds, these functions should not change much across muscle lengths. We found that in one muscle (LR in monkey 3, Fig. 4B) this is indeed the case, and a single reduced relaxation function could be used to fit all the steps. However, in the other three muscles there are considerable variations, which extend throughout our observation period. To more clearly highlight how *G(t)* varies as function of muscle elongation, in each panel in Fig. 4 we have added a small inset. Here we plot the value of *G(t)* at time 3.2 ms (indicated by a gray vertical line in each panel) as a function of muscle elongation. These deviations might not seem to be very large, but they are comparable to those observed in other tissues for which it was concluded that separability does not hold [36]. Also note that there is a smooth transition across elongations, which would not be expected if the variations were due to random noise (each relaxation response has been fit independently to a separate QLV model). Accordingly, we must conclude that even though we have been able to use the generalized QLV model to fit individual steps (by selecting a different set of parameters for each step), for most muscles there is no single *G(t)* that would allow the original QLV model to accurately reproduce all the step responses. Thus separability of the relaxation response does not appear to be a general property of passive eye muscles. Nonetheless, we feel that it is appropriate to provide an “average” QLV model (dashed gray lines in Fig. 4, Table 2). While this model neglects the differences that we just reported, it reproduces the step response of passive eye muscle with a level of accuracy that might be sufficient for most applications.

**Figure 5:**
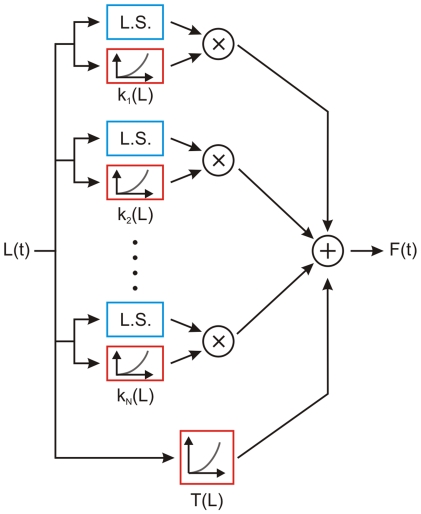
Schematic view of the adaptive QLV model (Eq. 17). Blue is used for linear processes, and red is used to indicate nonlinearities. Because the nonlinearity does not precede the linear stage (L.S.), superposition does not hold (unlike the QLV model, Fig. 1B).

**Table 2:** Parameters for the QLV model (average fit for each muscle).

	α	g(1.3 ms)	g(7.1 ms)	g(40 ms)	g(225 ms)	g(1.26 s)	g(7.11 s)	g(40 s)
m2SR	0.1251	0.2439	0.3408	0.1864	0.0787	0.0460	0.0454	0.0588
m3LR	0.0879	0.5867	0.1214	0.1199	0.0573	0.0395	0.0321	0.0431
m3SR	0.1794	0.2423	0.2246	0.2280	0.1051	0.0676	0.0613	0.0711
m4LR	0.1225	0.3034	0.2291	0.1935	0.0745	0.0731	0.0401	0.0864

### Applying the AQLV model to passive eye muscles

Nonlinear viscoelastic models that do not assume separability and rely instead on the more general integral equation [34], [35]

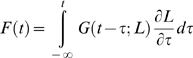
could reproduce the observed (i.e., not separable) behavior of EOMs. In our case, the generalized QLV model, which belongs to this class of models, does it quite well. However, this is also accomplished by a simpler (i.e., with a smaller number of parameters) and elegant model inspired by the QLV theory [36]. To understand how this model differs from the QLV model, let us now plug Eq. 5 into Eq. 7:
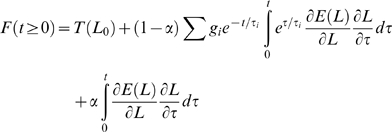
(15)


The first and last terms can be grouped together, as they essentially represent the steady-state elastic response at the current length, i.e., 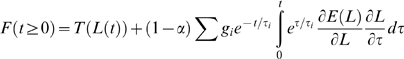
(16)


In the second term in this formula the model nonlinearity is embedded in the elastic response *E(L)*. Nekouzadeh and colleagues [36] proposed moving this dependency out of the integral, thus defining moduli that explicitly depend nonlinearly on length:
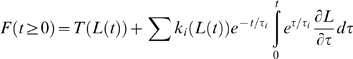
(17)


The moduli are here indicated with *k_i_* instead of *g_i_* to highlight their physical meaning: they essentially represent the glassy (i.e., dynamic) stiffness of the muscle. The authors called this model *adaptive quasi-linear* (or AQLV); we show its block diagram in Fig. 5.

**Figure 6:**
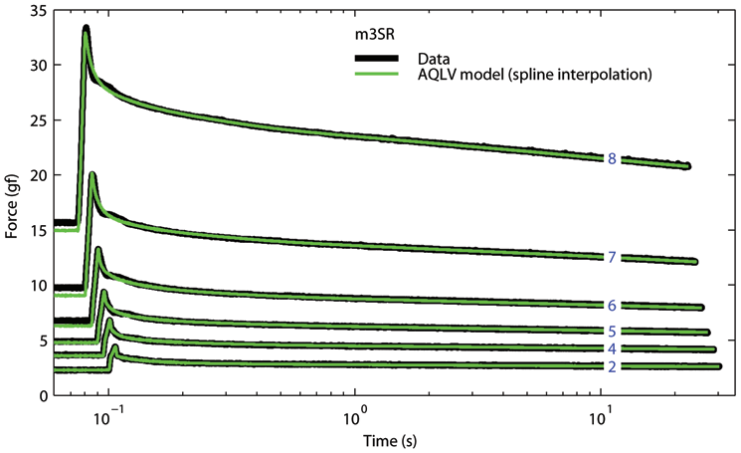
Step responses and AQLV model fits. Data (black) and force predicted by the AQLV model (green). The values for the parameters of the model at each step length were derived from the parameters of the generalized QLV model described above. A cubic spline interpolation (Fig. 7C) was then used to determine the value of the parameters at other lengths. Data for the superior rectus in m3. Each step has been offset in time for clarity. Note that in a logarithmic plot to carry out this operation without deforming the shape the time axis must be compressed, not shifted.

If we now proceed as we did above for the generalized QLV model, we can again estimate the parameters of the AQLV model from the fits that we presented in our preceding paper. With the AQLV model the force after one of our quick steps can be approximated with
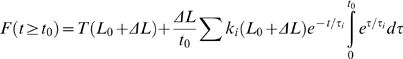
(18)


Equating Eqs. 6 and 18, we find that 
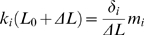
(19)


Again, δ_i_ (Eq. 10) would always be equal to 1 if our steps were instantaneous. Note that since δ_i_ is dimensionless, and *m_i_* has the dimension of a force, Eq. 19 is compatible with the interpretation of *k_i_* as a stiffness. Because this procedure for determining *k_i_* from *m_i_* rests on the same assumptions used to determine *g_i_* from *m_i_*, instead of using our original estimates of the moduli *m_i_*, we can use the values that we found when we optimized the moduli to yield the best fit to the peri-step data with the generalized QLV model. Naturally, as we did for the QLV model we had to also extend the AQLV model by adding the purely viscous term:
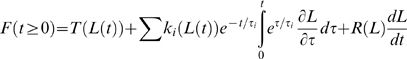
(20)


This led to excellent fits over all the steps tested, during both the peri- and post-elongation epochs. In Fig. 6 we show the fits to steps when the functions *k_i_(L)* are found by interpolating with a cubic spline function over the values (one for each step) obtained from Eq. 19. Because the spline does not extrapolate, the step executed from the shortest length could not be simulated; the fits for the other steps are, however, extremely good. Some small biases before the step are to be expected, as the muscle had not completely settled to its equilibrium force (a necessary assumption in all of our simulations).

**Figure 7:**
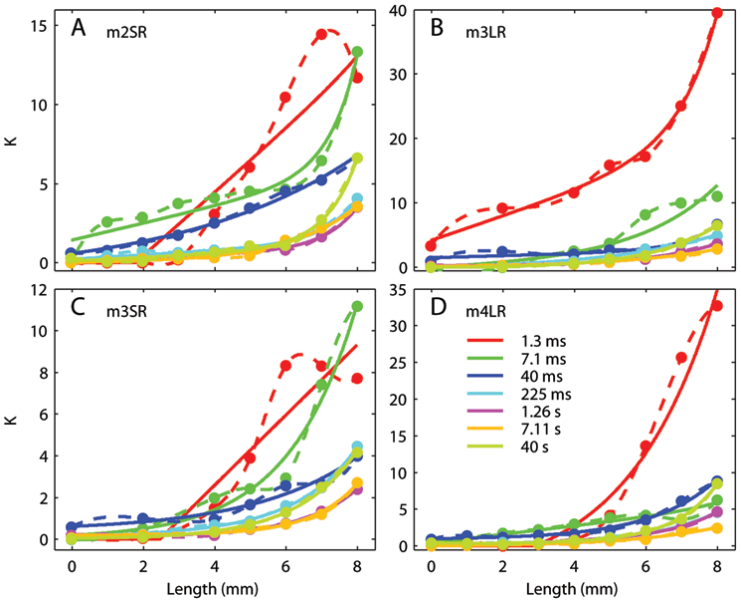
Parameters for the AQLV model. Each panel shows the parameters for a different muscle, as a function of length at the end of the step. The parameter values (dots) have been computed from the optimized QLV parameters, and the length shown is the muscle length after the end of the step. To estimate the values for an arbitrary length we then either used a cubic spline interpolation (dotted lines, used in Fig. 6), or a four-parameter nonlinear fit (solid lines, used in Fig. 8).

Next, we used the same parametric function that we proposed for the length-tension relationship (Eq. 4) to fit the dependence of each *k_i_* on length. This allows us to extrapolate the lower range, and thus also to simulate the first step. In Fig. 7 we plot *k_i_* as a function of length for each time constant, separately for each muscle. Both the spline interpolation (dashed, used in Fig. 6) and the linear-exponential fit (solid) are shown. In general the parametric fits are quite good, with the exception of the smallest time constant in m2SR and m3SR. We do not have any explanation for this difference, but when we manually raised the values for this time constant at the largest elongation, indicated by the rightmost red point in each panel, to 14 (for m2SR) and 20 (m3SR), so that a good parametric fit could be obtained, the model fit to the step at this elongation deteriorated only marginally (remember that at these large elongations the fit was not exceptional to start with, Fig. 3F).

**Figure 8:**
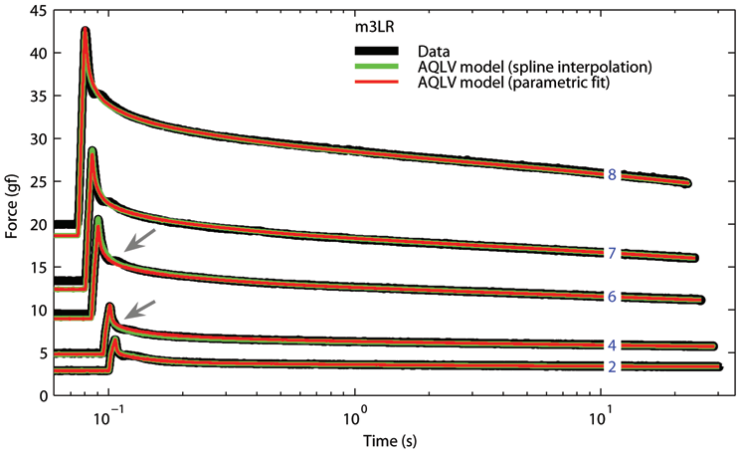
Step responses and AQLV model fits. Data (black) and force predicted by the AQLV model. A four-parameter non-linear equation was used to fit the values for the parameters derived from the QLV model at each step length (red trace). Because this model has fewer degrees of freedom than the cubic spline used in Fig. 6(and shown here in green), the fit is not as good (gray arrows point to the largest discrepancies between the two models), but it is very good nonetheless. Data for the lateral rectus in m3, the muscle for which we obtained the worst fit between model and data. Just as in Fig. 6, the traces are offset in time for clarity.

When we use the fitted function to simulate the force generated by the muscle the fits deteriorate somewhat (which is not surprising, as we have reduced the number of parameters from 70 to 35 in m2SR and m4LR, and from 56 to 35 in m3LR and m3SR), but they are still extremely good. In Fig. 8 we show the data from the muscle that exhibited the largest discrepancies between the two fit methods (highlighted by the gray arrows). In Tables 3–6 we list the values of the parameters of the fitted functions for each muscle.

**Table 3:** Parameters for the *k_i_(L)* functions in m2SR.

	1.3 ms	7.1 ms	40 ms	225 ms	1.26 s	7.11 s	40 s
a	2.0000	0.5341	0.0001	0.1342	0.1025	0.0000	0.1108
b	0.0002	0.0003	1.5688	0.0004	0.0004	0.0571	0.0006
c	0.9945	0.7906	5.0000	0.9038	0.9095	1.9272	0.8745
d	−3.55	1.43	−0.99	0.22	0.05	−0.03	0.10
R^2^	0.90	0.96	0.99	0.99	1.00	0.99	0.99

**Table 4 pone-0006480-t004:** Parameters for the *k_i_(L)* functions in m3LR.

	1.3 ms	7.1 ms	40 ms	225 ms	1.26 s	7.11 s	40 s
a	1.8726	0.1935	0.1557	0.0000	0.0002	0.0033	0.0053
b	0.0102	0.3000	0.0009	0.4418	0.0671	0.1375	0.0815
c	1.0534	2.1947	0.9559	3.1828	2.0185	2.6526	1.8191
d	4.20	−0.30	1.48	−0.38	0.14	−0.01	−0.14
R^2^	0.99	0.89	0.95	0.98	0.99	0.98	1.00

**Table 5 pone-0006480-t005:** Parameters for the *k_i_(L)* functions in m3SR.

	1.3 ms	7.1 ms	40 ms	225 ms	1.26 s	7.11 s	40 s
a	1.6818	0.0007	0.0002	0.0313	0.0001	0.0004	0.0001
b	0.0000	0.1719	0.2241	0.0361	0.0131	0.0043	0.0365
c	4.0502	1.9120	2.8916	1.6933	1.5487	1.2575	1.6803
d	−4.13	0.02	0.39	0.10	0.12	0.19	0.00
R^2^	0.89	0.98	0.96	1.00	1.00	0.99	1.00

**Table 6:** Parameters for the *k_i_(L)* functions in m4LR.

	1.3 ms	7.1 ms	40 ms	225 ms	1.26 s	7.11 s	40 s
a	0.0001	0.5696	0.0001	0.0001	0.1055	0.0000	0.0008
b	1.5364	0.0008	0.1115	0.0542	0.0145	0.1044	0.0119
c	2.4569	1.1318	1.8629	1.8236	1.4410	2.4816	1.2263
d	−5.00	0.44	0.88	0.25	0.07	−0.09	0.35
R^2^	0.97	0.93	0.99	0.99	0.99	0.97	1.00

### Testing the Superposition Principle: Double Steps

**Figure 9:**
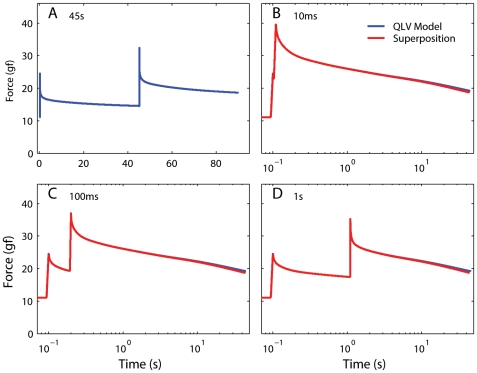
The QLV model obeys superposition. A: A sequence of two elongation steps (0.5 mm each), separated in time by 45 s, was simulated using the generalized QLV model, and the resulting force is plotted against time. B: The same two steps shown in A are applied, but now the temporal separation (ISI) is only 10 ms. (Blue: Model output. Red: Output expected if the superposition principle is obeyed. Note logarithmic scale for time.) C: Same as in B, but with an ISI of 100 ms. D: Same as in C, but with an ISI of 1 s. In all cases the model output matches the superposition prediction. The small deviation between the two traces toward the end of each simulation is due to the incomplete settling of the model output just before the second step in panel A (on which the superposition prediction is based).

The second assumption in Fung's model is that the forces induced by two subsequent elongation changes are independent, and thus sum linearly (i.e., superposition holds). In Fig. 9 we plot the force predicted by the optimized generalized QLV model described above in response to a sequence of two steps (the model parameters were those that best fit the second step in the sequence in m4LR). We used four sequences of two steps, each with a different inter-step interval (ISI, 10 ms, 100 ms, 1 s, and 45 s). In all cases the initial length and the step amplitudes were identical. We assumed that with an ISI of 45 s (Fig. 9A) the force induced by the second step is independent of the first step. We indicate with t_A_ the starting time for the first step, and with t_B_ the starting time for the second step. We then take the change in force from time 0 up to the second step as a template for the force induced by the first step, i.e., 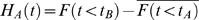
and the change in force from 1 s before the second step to the end of the recording as a template for the force induced by the second step
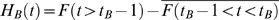



Each template thus measures the change in force induced by the step over approximately 45 s, and it is shifted in time so that *H(t_0_)* is the change in force at the end of each step (*t_0_* is the step duration). It thus follows that *H(t<0)* is essentially zero. Figure 9B shows (in blue) the model output when the same steps shown in Fig. 9A are simulated with an ISI of 10 ms. In red we show the prediction from superposition, i.e., 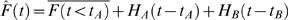



Because *H_A_* does not extend far enough, this predicted trace terminates slightly before the model output trace. In panels C and D we show the results for ISIs of 100 ms and 1 s. As expected, in all cases the superposition prediction matches the model output very well. The small deviation between the two traces toward the end of each simulation is due to the incomplete settlement of the model output just before the second step in panel A, which causes *H_B_* to increasingly (but only slightly) underestimate the force induced by the second step.

**Figure 10:**
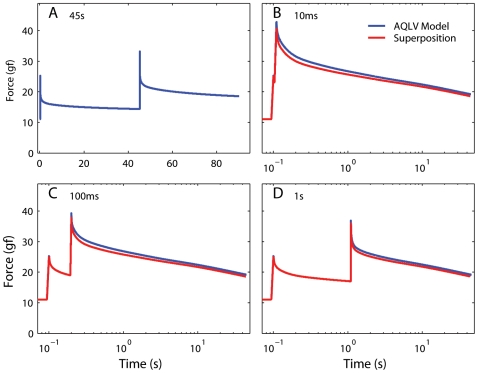
The AQLV model does not obey superposition. Same as Fig. 9, but now the AQLV model is used for the simulations. In all cases the model output is larger than the superposition prediction. The deviation between the two traces is maximal at the end of the second step, and is larger for shorter inter-step intervals. Some of the deviation toward the end of each simulation can be imputed to the incomplete settling of the model output just before the second step in panel A, but the initial deviation is due to the lack of superposition in this model.

In the AQLV model the nonlinearity does not precede the linear stage anymore (Fig. 5), and thus superposition does not hold. More precisely, because the stiffness parameters for the AQLV model increase with length (shown in Fig. 7 for our data set, but this is a general behavior), if two steps are executed one after the other, this model predicts that the force generated by the second step will be *larger* than that predicted by the superposition principle. We repeated the same simulations that we carried out with the generalized QLV model, using as parameters for the AQLV model those that best matched the m4LR. As expected, at all ISIs the model output is larger than the superposition prediction, with the maximal deviation occurring at the end of the second elongation step (Fig. 10).

**Figure 11:**
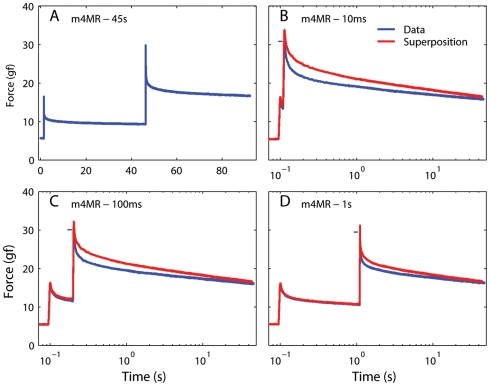
Testing the superposition hypothesis in muscle at long lengths. A: A sequence of two elongation steps (0.5 mm each), separated in time (ISI) by 45 s, was applied, and the resulting force measured. B: The same two steps shown in A are applied, but now the ISI is only 10 ms. (Blue: Force measured. Red: Force predicted by the superposition principle) C: Same as in B, but with an ISI of 100 ms. D: Same as in C, but with an ISI of 1 s. For clarity, the maximum force recorded is marked by a small horizontal blue bar just to the left of the value. In all cases the prediction is initially considerably higher than the actual force, indicating that the superposition principle does not hold in muscle at long lengths.

To test whether either model predicts the actual behavior in passive EOMs, we applied the very same sequences of two steps simulated above to two extraocular muscles. In each set of sequences the initial muscle length and the step amplitudes were identical. In Fig. 11 we show the results of this experiment in the medial rectus of m4 at large elongations. The same conventions used in Figs. 9 and 10 apply, but now the blue line indicates the force actually induced in the muscle instead of a simulation output. In all cases the force measured was significantly *smaller* that the force predicted by the superposition principle (red traces).

**Figure 12:**
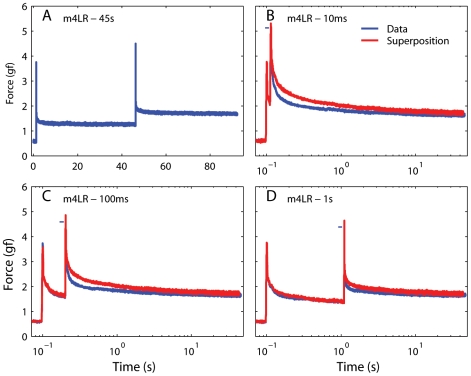
Testing the superposition hypothesis in muscle at short lengths. Same as Fig. 11, but in a different muscle and at the low end of the elongation range (notice the much smaller forces). Superposition does not hold at short lengths either.

This experiment was performed at the limit of our elongation range, a region often described as being “more nonlinear”. Some experiments have suggested that the quasi-linear model might hold up better, at least as far as the separability is concerned, at short elongations [48]. To test the possibility that this might be true also for superposition, we ran this same experiment at the low end of the elongation range tested. Figure 12 illustrates the results obtained in the lateral rectus of m4. In this case the steps were executed within a range of elongations where the length-tension curve can be fit with a straight line [37]. Obviously, the forces in play are much smaller than those shown in Fig. 11, and the S/N ratio is also lower, but the result is the same: the force induced by the second step is significantly smaller than that predicted by superposition. Superposition failed in this manner in all cases tested (two muscles, two initial elongations per muscle). As shown above, neither the original nor the adaptive QLV models can reproduce this behavior (the latter actually performed worse than the former on this experiment).

## Discussion

### The quasi-linear theory and its extensions

Fung's QLV theory has enjoyed great success for over three decades. This success must be attributed in large part to its ability to account for many experimental observations, but substantial contributors to this success were also its undeniable elegance, and the limited experimental burdens it imposed. We have shown here that in passive extraocular muscles the two basic assumptions of the QLV theory do not hold: relaxation response after a step does not scale with length (i.e., it is not separable), and two successive elongations interact, so that the superposition principle does not hold.

Recent studies in other tissues had also come to the conclusion that separability might not be typical of passive biological tissues [25], [32]–[36], although other researchers proposed that this property might hold, but only for small elongations (up to approximately 15% of the resting length) [48]. To rectify this failure of the QLV theory to deal with the data, an extension to the original quasi-linear model was recently proposed [36], yielding a model (AQLV) that accounts for the step responses reported here (Figs. 6 and 8). Unfortunately this model cannot reproduce the results of our double-step experiments, actually underperforming the original QLV model in these tests. Because the force induced by a step of elongation increases nonlinearly with length, all models that rely on the general formulation [35]

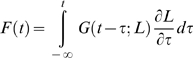
(21)will similarly be unable to account for our data.

### Lack of superposition: implications for motor control

**Figure 13:**
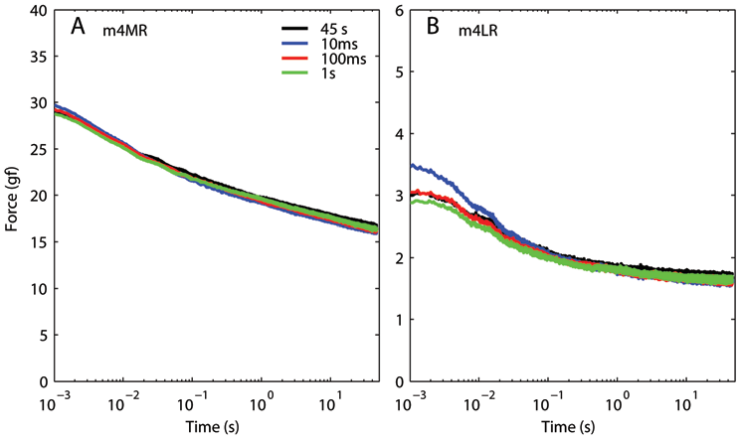
Force induced by the second step in a double-step sequence. A: Data from elongations at long lengths, same dataset as in Fig. 11 . The force induced by the second elongation step is *not* a function of the ISI. B: Data from elongations at short lengths, same dataset as in Fig. 12. With the exception of the first 20 ms after the shortest ISI, the force induced by the second elongation step is invariant. The muscle thus appears to have no memory of the previous elongation.

From an experimental point of view, the most important result that we have presented in this paper is that, for passive extraocular muscles in monkeys, the principle of superposition does not hold. More precisely, the force measured after the second step in a sequence is lower than that predicted by superposition. To better evaluate the potential significance of this finding, in Fig. 13 we plot the force induced by the second step in each sequence of double steps. Strikingly, it appears that the force is essentially always the same, regardless of the preceding movement. The peak force is higher as the ISI decreases, even though less than predicted by superposition (see Figs. 11 and 12). However, after 20 ms or so the traces are virtually indistinguishable. For ISI of 100 ms or more, the traces are for all practical purposes identical. Note that in physiologic conditions sequences of eye movements are always separated by at least 100 ms. In this sense, it is then tempting to conclude that passive extraocular muscles have essentially no memory: the force they generate is only a function of the last elongation to which they have been subjected, and it does not depend on the previous history. Needless to say this might simplify the motor control problem, as the neural controller would not have to keep track of the muscle's elongation history. Given the limited amount of data that we have collected, at this point this must be considered a speculation, but we believe that it is a speculation worthy of further investigation.

### Limits of our experimental approach

Our minimally invasive approach was the only one consistent with our goal of obtaining measurements that could be directly incorporated in a model of the eye plant to be used for diagnostic assistance. However, our *in vivo* preparation imposed several constraints on the elongations that we could impose, on the stiffness of the apparatus, on the signal to noise ratio, and on the bandwidth of our measurements. Furthermore, since some of the muscles that we pulled on were partially wrapped around the eyeball, it is conceivable that translations and/or deformations of the eyeball could have affected our measurements (they would essentially be equivalent to an increased compliance of the apparatus). Strictly speaking, the force we report here is thus the force that would be applied on the eyeball by a passive antagonist muscle when it is extended by the action on the globe of a shortening agonist muscle. Because of the presence of the eyeball, the elongation of the passive muscle could be somewhat smaller than the motion of its insertion on the eyeball.

While simulations show that the stiffness of the apparatus did not significantly affect our measures, and we are confident that significant translations of the globe did not occur, it is harder to rule out small deformations of the (unobservable) posterior pole of the eyeball. Nevertheless, there is no reasonable scenario under which these potential artifacts could have affected *qualitatively* the findings here reported. In particular, they cannot be responsible for the relationship between step response and muscle elongation in the generalized QLV model (Fig. 4, insets), and they certainly cannot affect the superposition of forces. It is worth noting that this last result came also from a medial rectus muscle, which does not wrap around the globe, and thus cannot induce translations or deformations of the eyeball. Finally, as we previously mentioned [37], across muscles there are significant quantitative differences, certainly larger than those that could have been induced by our less-than-ideal preparation. The data we report here should thus be perfectly appropriate to build a model of an average passive eye muscle.

While we are confident that none of these limitations qualitatively affects our results, it is obvious that *ex-vivo* preparations could provide more accurate results. Since other laboratories are better equipped for carrying out *ex-vivo* experiments, it is our hope that our results will stimulate their interest. In particular, it would be very interesting to know whether the lack of superposition that we have reported holds also at the single fiber level (in extraocular or skeletal muscles), and whether it is a general feature of passive biological tissues.

### Modeling our data

Now that we have shown that the quasi-linear theory, and in general models that can be formulated as a single integral, cannot account for our data, it is natural to ask what other theory could. One classic way of performing nonlinear system identification is to build a non-parametric, input/output description of the system (i.e., the system is seen as a black-box, without any attempt to describe what is inside the box). For example, a Volterra series approximation, analogous to the Taylor series approximation of a function, yields a description of the system based on a series of functionals [49]. Unfortunately the Volterra kernels are not orthogonal to each other (i.e., in the response, the n-th order interaction of the previous inputs is described by all kernels of order n or higher), and thus their estimation requires the simultaneous solution of a set of integral equations. In practice, a modified series must be used. Wiener showed that if the input signal is a white-noise Gaussian signal, a series of orthogonal kernels can be derived [50], making their estimation considerably simpler. This technique is commonly used in many fields, but it has a major limitation in our specific case: we can only measure positive forces, i.e., the inputs that stretch and relax the muscle are not symmetric. Thus, a simple Gaussian white-noise input signal governing length could not be used to identify the Wiener kernels for muscle.

In the context of nonlinear viscoelastic behavior, a model that follows this same philosophy has been proposed by Pipkin and Rogers [51]. This model has two major advantages over the Wiener kernels: first, it only requires the measurement of the force induced by elongation steps and sequences of elongation steps. Second, the first term in the series is a single integral with a nonlinear integrand (Eq.21), and it would thus be possible to see this model as an extension of Fung's model (or more precisely of the AQLV model, which would then become the first order approximation of the overall model). The second term in the series is then computed by looking at the difference between the force predicted by this first term and the actual response to sequences of two steps, and so on. However, this theoretical elegance does not translate into an actual ease of implementation. Vast quantities of data must still be collected; for example, all delays between the pairs of steps must be tested, and this must be done separately at each length. Furthermore, it is far from trivial to obtain the expressions for the series terms past the first (to quote Pipkin and Rogers: “For the case n = 2 we have deduced the proper form [for the second term], but the analysis is lengthy, and we omit it.”). Another disadvantage of this technique (and of all non-parametric techniques in general) is that even a “simple” nonlinear model might require many terms. For example, a Hammerstein system (the cascade of a static nonlinearity and a linear dynamical system, like the original QLV model) with a squaring nonlinearity, has an infinite zeroth-order Weiner kernel [52], [53]. Thus, the non-parametric approach guarantees a solution, but not a simple or easy to compute solution, even when one exists.

An alternative to this nonparametric approach is to use a parametric model, in which the general structure of the model is first guessed based on the current knowledge of the system. Then, an experiment is designed to probe that structure as extensively as possible. Finally, an optimization algorithm is used to identify the parameters by fitting the model to the data collected [54]. Needless to say, if the initial assumptions turn out to be incorrect, this approach inevitably leads to a dead end.

There is also a third alternative. As the model proposed by Nekouzadeh and colleagues [36] does a good job at fitting the single step data (although admittedly with a lot of parameters, 35 in our case), and to a lesser extent so does the original QLV model (with 8 parameters), it is conceivable that one could build on it by manipulating its output to also fit the double step data. Of course there are many different ways to accomplish this feat, and other experimental paradigms will then be needed to validate the resulting model. In the next paper in this series we will proceed along these lines.
